# Partial mGlu_5_ Negative Allosteric Modulator M-5MPEP Demonstrates Antidepressant-Like Effects on Sleep Without Affecting Cognition or Quantitative EEG

**DOI:** 10.3389/fnins.2021.700822

**Published:** 2021-07-02

**Authors:** Kimberly M. Holter, Alex D. Lekander, Christina M. LaValley, Elizabeth G. Bedingham, Bethany E. Pierce, L. Paul Sands, Craig W. Lindsley, Carrie K. Jones, Robert W. Gould

**Affiliations:** ^1^Department of Physiology and Pharmacology, Wake Forest School of Medicine, Winston-Salem, NC, United States; ^2^Wake Forest University, Winston-Salem, NC, United States; ^3^Department of Pharmacology, Vanderbilt University Medical Center, Nashville, TN, United States; ^4^Warren Center for Neuroscience Drug Discovery, Vanderbilt University, Nashville, TN, United States; ^5^Department of Chemistry, Vanderbilt University Medical Center, Nashville, TN, United States

**Keywords:** electroencephalography (EEG), cognition, metabotropic glutamate receptor 5 (mGlu5), negative allosteric modulator (NAM), MK-801

## Abstract

Selective negative allosteric modulators (NAMs) targeting the metabotropic glutamate receptor subtype 5 (mGlu_5_) demonstrate anxiolytic-like and antidepressant-like effects yet concern regarding adverse effect liability remains. Functional coupling of mGlu_5_ with ionotropic *N*-methyl-D-aspartate receptors (NMDARs) represents a potential mechanism through which full inhibition leads to adverse effects, as NMDAR inhibition can induce cognitive impairments and psychotomimetic-like effects. Recent development of “partial” mGlu_5_ NAMs, characterized by submaximal but saturable levels of blockade, may represent a novel development approach to broaden the therapeutic index of mGlu_5_ NAMs. This study compared the partial mGlu_5_ NAM, M-5MPEP, with the full mGlu_5_ NAM, VU0424238 on sleep, cognition, and brain function alone and in combination with a subthreshold dose of the NMDAR antagonist, MK-801, using a paired-associates learning (PAL) cognition task and electroencephalography (EEG) in rats. M-5MPEP and VU0424238 decreased rapid eye movement (REM) sleep and increased REM sleep latency, both putative biomarkers of antidepressant-like activity. Neither compound alone affected accuracy, but 30 mg/kg VU0424238 combined with MK-801 decreased accuracy on the PAL task. Using quantitative EEG, VU0424238, but not M-5MPEP, prolonged arousal-related elevations in high gamma power, and, in combination, VU0424238 potentiated effects of MK-801 on high gamma power. Together, these studies further support a functional interaction between mGlu_5_ and NMDARs that may correspond with cognitive impairments. Present data support further development of partial mGlu_5_ NAMs given their potentially broader therapeutic index than full mGlu_5_ NAMs and use of EEG as a translational biomarker to titrate doses aligning with therapeutic versus adverse effects.

## Introduction

Functional antagonism of the metabotropic glutamate receptor subtype 5 (mGlu_5_) represents a promising target with broad therapeutic potential for the treatment of numerous disorders including fragile X syndrome (Yan et al., 2005; Dölen et al., 2007; for review see Nickols and Conn, 2014), Parkinson’s disease (Morin et al., 2010), anxiety (Busse et al., 2004; Swanson et al., 2005), depression (Lindemann et al., 2015), acute and neuropathic pain (Montana et al., 2009; Cavallone et al., 2020), and substance use disorder (McGeehan and Olive, 2003; Yararbas et al., 2010; Veeneman et al., 2011; Gould et al., 2015). Moreover, mGlu_5_ is involved in homeostatic sleep regulation (Weigend et al., 2019; Aguilar et al., 2020) and modulation of mGlu_5_ has potential to normalize sleep disturbances associated with a number of the aforementioned conditions (Lindemann et al., 2015; Gould et al., 2017). For example, in major depressive disorder (MDD), commonly reported sleep disturbances in patients include reductions in rapid eye movement (REM) sleep latency and increased REM duration/density (Armitage, 2007; Steiger and Pawlowski, 2019). In rodents, basimglurant, a full mGlu_5_ negative allosteric modulator (NAM), decreased REM duration and increased REM sleep latency (Lindemann et al., 2015), an EEG profile similar to several clinically prescribed antidepressants (Wichniak et al., 2017; Steiger and Pawlowski, 2019). Despite strong evidence for therapeutic potential of mGlu_5_ antagonism, an early human study reported dissociative-like effects in patients administered fenobam, introducing concerns surrounding the viability of this mechanism (Pecknold et al., 1982). Subsequently, preclinical studies examining several first generation full mGlu_5_ NAMs including MTEP and fenobam reported sedation, psychotomimetic-like effects, and cognitive impairments (Kinney et al., 2003; Homayoun et al., 2004; Jacob et al., 2009; Abou Farha et al., 2014; Gould et al., 2015). Thus, broadening the therapeutic index and developing translational methods for reliably predicting dose-effect relationships is critical for successful development of mGlu_5_ NAMs.

Development and characterization of partial mGlu_5_ NAMs has introduced the novel idea that submaximal inhibition of mGlu_5_ may be sufficient to engender therapeutic effects while mitigating adverse effect liability (Gould et al., 2015, 2017). The partial mGlu_5_ NAMs M-5MPEP and Br-M5MPEPy produced approximately 50% inhibition of the maximal glutamate response *in vitro* at concentrations that fully displaced [3^H^]methoxyPEPy binding at the allosteric site (Rodriguez et al., 2005). *Ex vivo* studies confirmed >85% receptor occupancy at these relevant dose ranges (Gould et al., 2015). Importantly, these partial mGlu_5_ NAMs exhibited comparable anxiolytic- and antidepressant-like activity as the full mGlu_5_ NAM, MTEP (Rodriguez et al., 2005; Gould et al., 2015).

There is also evidence suggesting partial mGlu_5_ NAMs display lesser potential for adverse effects. In addition to coupling to G_q/__11_ proteins and inducing downstream calcium mobilization, mGlu_5_ is physically and functionally coupled to the ionotropic *N*-methyl-D-aspartate receptor (NMDAR) (Shigemoto et al., 1993; Pisani et al., 2001; Marino and Conn, 2002; Niswender and Conn, 2010; O’neill et al., 2018). Downstream inhibition of NMDARs represents one hypothesized mechanism underlying adverse effects associated with full mGlu_5_ NAMs (for review see Sengmany and Gregory, 2016). NMDAR inhibition is known to induce cognitive impairments and psychotomimetic-like effects in humans and in animals, typically measured via hyperlocomotion (Morris et al., 1986; Krystal, 1994; Homayoun et al., 2004; Talpos et al., 2015). Interestingly, unlike the full mGlu_5_ NAM MTEP, these partial NAMs did not potentiate hyperlocomotion induced by phencyclidine (PCP), an NMDAR antagonist (Gould et al., 2015). In the present study, we sought to further investigate the possible antidepressant-like and adverse effects associated with partial versus full inhibition of mGlu_5_ and the functional interaction with NMDARs.

Using translational methods to assess cognition and brain function, we examined effects of M-5MPEP and VU0424238, a full mGlu_5_ NAM demonstrating anxiolytic- and antidepressant-like activity and ∼95% maximal glutamate inhibition (Felts et al., 2017). First, M-5MPEP or VU0424238 were examined on sleep architecture and brain function using electroencephalography (EEG) in freely moving rats. Changes in sleep/wake latency and durations (wake; Rapid Eye Movement, REM; or Non-REM, NREM sleep) as well as spontaneous locomotor activity were simultaneously measured to examine if M-5MPEP and VU0424238 had sleep-altering profiles similar to clinically prescribed antidepressants. Next, each compound was examined on working memory performance in rats trained to perform a paired-associates learning (PAL) task. The PAL task, adapted from the human PAL task in the CANTAB battery, utilizes a touchscreen platform to assess cognitive function through object-location learning and is sensitive to glutamatergic manipulations (Taffe et al., 2002; Day et al., 2003; Robbins and Murphy, 2006; Talpos et al., 2009, 2014; Horner et al., 2013). Lastly, we sought to investigate the functional interaction between mGlu_5_ and NMDARs using quantitative EEG (qEEG). Changes in high frequency gamma band power represent a highly translational biomarker of brain function that can be examined in animals and humans (Steiger and Kimura, 2010; English et al., 2014; Javitt et al., 2020). Recently, changes in gamma band power have been used in preclinical studies to predict the degree of NMDAR antagonism that produced antidepressant effects without disrupting cognition in humans (Sanacora et al., 2014). Herein, we employed a similar biomarker approach to assess the effects of M-5MPEP or VU0424238 in combination with a subthreshold dose of MK-801, an NMDAR antagonist, on PAL performance and gamma band power. We hypothesized that M-5MPEP and VU0424238 alone would not affect PAL performance within dose-ranges known to produce anxiolytic- and antidepressant-like effects. Further, we hypothesized that VU0424238 but not M-5MPEP would disrupt cognition and potentiate gamma power in combination with a subthreshold dose of MK-801.

## Materials and Methods

### Subjects

Twenty-seven male Sprague-Dawley rats (250–275 g; Envigo, Indianapolis, IN, United States) were pair housed in opaque cages (8 in × 10 in × 18 in). Twelve pair housed rats were initially trained for cognition studies and were maintained at 85% of their free-feeding weights with *ad libitum* access to water. Fifteen separate rats were individually housed following implantation of EEG transmitters and had *ad libitum* access to food and water. All rats were maintained on a 12 h light/12 h dark cycle and were housed in a temperature (range: 70–80°F) and humidity-controlled (range: 30–70%) colony room. Female rats were excluded from this initial study due to complexities regarding known estrogen receptor interactions with mGlu_5_ and metabolic differences affecting response to NMDAR antagonists (Nabeshima et al., 1984; Hönack and Löscher, 1993; Wessinger, 1995; Meitzen and Mermelstein, 2011; Feinstein and Kritzer, 2013). All animal care procedures were approved by the Wake Forest University Animal Care and Use Committee and complied with the National Institutes of Health guide for the care and use of Laboratory animals.

### Drugs and Reagents

M-5MPEP (18, 30, 56.6 mg/kg, ip) and VU0424238 (1, 3, 10, 30 mg/kg, ip) were synthesized by Vanderbilt University within the Center for Neuroscience Drug Discovery and the Institute for Chemical Biology as previously described (Rodriguez et al., 2005; Felts et al., 2017). Both compounds were formulated in 10% Tween 80 in saline as microsuspensions. (+) MK-801 hydrogen maleate (0.1, 0.18, 0.3 mg/kg sc; Sigma Aldrich, St. Louis, MO, United States) was formulated in sterile saline as an aqueous solution. All compound formulations were adjusted to a pH of 6-7. Compound administration followed a within-subject, counter-balanced design with a minimum of a 3 days (cognition) or 5 days (EEG) washout period between test sessions. The selected dose ranges have been previously shown to engender behavioral effects in rodents including anxiolytic-like and antidepressant-like effects as well as the ability to decrease cocaine self-administration. Specifically, 18 and 56.6 mg/kg M-5MPEP correspond with ∼50 and ∼80% *ex vivo* receptor occupancy, with 18 and 32 mg/kg producing maximal effects in decreasing immobility duration in the forced swim test and decreasing the number of marbles buried in a marble-burying assay, respectively (Gould et al., 2015). 1 and 10 mg/kg VU0424238 correspond with ∼50 and ∼80% *in vivo* receptor occupancy, and dose-dependently decreased immobility duration in the forced swim task and decreased the number of marbles buried in the marble-burying assay with a maximal effect at 30 mg/kg (Felts et al., 2017).

### Paired-Associates Learning (PAL) Task

#### Touchscreen Training

Rats were trained 5 days per week during the first half of their dark (active) cycle in operant chambers (Lafayette Instruments, Lafayette, IN, United States) to respond to stimuli presented on a touchscreen. Stimuli appeared on the screen after rats broke an infrared beam in close proximity to the stimuli with a nose-poke (Horner et al., 2013; Talpos et al., 2015). First, rats learned to locate and consume 0.2 mL of diluted Ensure^®^ (33% mixed in water) delivered via a peristaltic pump into a receptacle on one wall of the chamber. Next, rats were trained to associate a visual stimulus with a reward delivery. A large white rectangle was presented on the entire screen. A nose-poke to the screen (or 30 s elapsed, whichever occurred first) resulted in stimulus removal, and reward delivery paired with illumination of a light within the reward receptacle and presentation of a 1-s tone. Following reward retrieval, the receptacle light was turned off and a 20 s inter-trial interval (ITI) was initiated. In the next sessions, rats were required to register a response on the screen to initiate reward delivery. Trial availability was signaled by illumination of a light within the receptacle and rats had to initiate each trial. Registering a nose-poke in the illuminated receptacle would extinguish the light, deliver a single click sound, and present stimuli on the touchscreen. Lastly, once each trial was initiated, a vertical white rectangle would appear in 1 of 3 stimulus locations on the screen. A nose-poke on the stimulus would result in trial termination and reward delivery. A nose-poke to a blank location on the screen terminated the trial resulting in removal of the stimulus from the screen and a 5-s time out with the house light on. The following trial after a blank touch was considered a correction trial (CT), which presented the stimulus again in the same location. CTs persisted until the rat made a correct response, which resulted in a reward and did not count toward the number of total trials initiated. Each session was terminated after 60 trials were completed or 60 min elapsed. Rats were required to complete 40 or more trials per training stage to progress to the next stage. Touchscreen training was complete when a rat performed at or greater than 80% accuracy (tracking a stimulus) on the final stage.

#### PAL Task Acquisition and Testing

In this visuospatial memory task, rats learn to associate one of three stimuli (spider, flower, or triangle) with a specific location on the screen. During each trial, two images are presented, one in the correct location and one in an incorrect location. Rats learn through trial and error to respond on the stimulus that is presented in its correct location to receive a reward. The present study employed the “dPAL” version of this task in which two “different” stimuli are presented (Talpos et al., 2009). At the beginning of each session, a reward was delivered and the magazine light turned on allowing the rat to consume the reward, nose poke, and initiate the first trial. A correct response resulted in reward delivery, illumination of the receptacle light, and a 1-s tone. A head entry into the receptacle initiated a 20 s ITI. Following an incorrect response, the screen went blank and the house light was illuminated for 5-s followed by CTs until a correct response was emitted. CTs were not counted toward the total number of trials or percent correct, but were counted as an additional dependent variable. Sessions terminated after 50 trials were completed or 60 min elapsed. Rats were considered to have acquired this task when performing at or above 70% accuracy after having completed greater than 40 trials for 5 consecutive days.

Following acquisition of the dPAL task, effects of the partial mGlu_5_ NAM M-5MPEP (vehicle 18, 30, 56.6 mg/kg; ip) and the full mGlu_5_ NAM VU0424238 (vehicle, 1, 3, 10, 30 mg/kg; ip) on performance were determined. Dose-response curves for mGlu_5_ NAMs alone were determined twice in a within-subject, counter-balanced design. If responding was below 10 trials following the first test day of VU0424238, a third determination was conducted. Rats were run 5–7 days per week and testing occurred ∼2 times per week with a minimum of 72 h between test sessions. Prior to the next test day, we ensured performance returned to baseline levels. Lastly, effects of NMDAR antagonist MK-801 (saline, 0.1, 0.18, 0.3 mg/kg; sc) were determined (once in each rat). We then examined effects of M-5MPEP (30 and 56.6 mg/kg) and VU0424238 (30 mg/kg) in combination with 0.1 mg/kg MK-801 (once in each rat). When examined alone, mGlu_5_ NAMs were administered 30 min prior to cognitive testing. For combination studies, mGlu_5_ NAMs were administered 30 min prior to MK-801, all test sessions were initiated 30 min after administration of MK-801.

#### PAL Task Analysis

To assess performance, number of selection trials per session, percent correct [(correct trails/number of selection trials) × 100], and percent of total trials that were CTs [(number of CTs/number of selection trials + number of CTs) × 100] were assessed. The percent of total trials that were CTs were examined in order to account for rats that did not complete all 50 selection trials (see Supplementary Figures). Values for each individual were averaged across both test days for each dose. Separate mixed-effects analyses of variance (ANOVAs) were applied to measure main effects of each compound with significance set at *p* < 0.05. If significant, Tukey’s multiple comparison *post hoc* tests were employed to compare each dose to all other doses. Test sessions during which an animal completed less than 10 selection trials were excluded.

### Examining Sleep/Wake Architecture and qEEG

#### Surgery

All animals were surgically implanted under isoflurane anesthesia and aseptic conditions with a telemetric transmitter (HD-S02; Data Sciences International [DSI], Minneapolis, MN, United States) for the wireless recording of EEG, electromyography (EMG), and motor activity as previously described (Nedelcovych et al., 2015; Gould et al., 2016, 2020). Transmitters were implanted subcutaneously just off the midline of the dorsal flank of each animal and leads were tunneled subcutaneously to the skull. Holes were drilled in the skull at +2 mm anterior to Bregma and +2 mm from the midline (frontal cortex) and at –6 mm posterior to Bregma and –2 mm from midline (contralateral occipital cortex). Exposed wires were placed directly in contact with the dura and secured via dental cement (Butler Schein, United States). In all animals, an additional set of leads were placed bilaterally in the nuchal muscle for EMG recording. Animals were individually housed following surgery for the duration of the study and recovered in the recording room for a minimum of 7 days.

#### EEG Recordings

For all studies, EEG, EMG and activity were recorded from the home cage of each animal continuously for 24 h beginning at the onset of the light cycle (Zeitgeber time 0; ZT 0) on the day of each study. Signals are wirelessly transmitted to a receiver placed beneath the homecage for offline analysis. In eight rats, M-5MPEP (Vehicle, 18, 30, 56.6 mg/kg, ip), VU0424238 (Vehicle, 3, 10, 30 mg/kg, ip), or MK-801 (0.1 mg/kg sc; based on cognition studies above) was administered 2 h after light onset to determine effects on sleep architecture and brain function. Using a within-subject design, each dose was tested once in a pseudorandom order and completed before switching compounds; test sessions occurred in all eight rats simultaneously. MK-801 was tested last. After initial dose-response curves were determined, effects of M-5MPEP (30 and 56.6 mg/kg) and VU0424238 (30 mg/kg) on MK-801-induced changes (0.1 mg/kg) were evaluated. For these combination studies, mGlu_5_ NAMs were administered 2 h into the light cycle and MK-801 was administered 30 min after administration of the mGlu_5_ NAM. In a separate group of seven rats, effects of the mGlu_5_ NAMs (56.6 mg/kg M-5MPEP and 30 mg/kg VU0424238) in combination with MK-801 were administered 2 h into the dark cycle (see Supplementary Material).

#### Sleep Staging and Analysis

Trained observers, blinded to treatment condition scored each 10-s epoch using Neuroscore 3.0 software (DSI) to determine sleep/wake stages, including Wake, NREM, REM sleep or artifact based on accepted characteristic oscillatory patterns (Nedelcovych et al., 2015; Gould et al., 2016, 2020). Artifact accounted for <1% of total recording time and was characterized by signal dropout. Artifact epochs were excluded from sleep and qEEG analyses. The amount of time in each stage (wake, NREM, REM) summed in 1 h bins across a 24 h period, the 4 h immediately after compound administration, and the 12 h dark cycle served as primary dependent measures. Additionally, latency to the first NREM and REM bout of a minimum of 20 s were quantified following mGlu_5_ NAM administration. Separate two-way analysis of variance (ANOVA) were applied to examine the effects of time and condition (dose) within each stage (wake, NREM, REM) during the 1 h bins. Separate repeated measure one-way ANOVAs were applied to examine the sum of each stage in 4 and 12 h bins and sleep latencies. When significant, a Dunnett’s *post hoc* test was performed. In all cases significance was defined as *p* < 0.05.

#### qEEG Spectral Power Analysis

Following sleep staging, quantitative EEG (qEEG) relative power spectra were computed in 1 Hz bins from 0.5 to 100 Hz using a Fast Fourier Transform with a Hamming window and overlap ratio of 0.5 within each 10 s epoch (Neuroscore, DSI). Relative power within each pre-defined frequency band (Delta [0.5–4 Hz], Theta [4–8 Hz], Alpha [8–12 Hz], Sigma [12–16 Hz], Beta [16–24 Hz], Low Gamma [30–50 Hz], High Gamma [50–100 Hz]) were subsequently separated by state (wake, NREM or REM), to examine state-dependent changes in power (Leiser et al., 2014; Gould et al., 2016). This study primarily focused on changes in power across each band during wake as well as delta power specifically during NREM sleep. Custom MATLAB scripts averaged data for each frequency band in 10-min bins for 7 h (2 h pre- and 5 h post-administration). Within-session changes are expressed as a percent change from each individual rat’s averaged 90-min baseline period directly prior to compound administration. These individual changes were then averaged to generate group effects. Additionally, to show changes across the entire power spectrum, all 10-s epochs in which each rat was awake were averaged in 1 Hz bins across the 5 h post-dosing period and expressed as a percent change from the 90-min baseline. Lastly, total activity counts were summed in 10-min bins for the 2 h baseline period and 5 h period following compound administration. Statistical analyses for dose-effect relationships within each band as well as the activity in 10-min bins were performed by mixed effects two-way ANOVAs followed by Dunnett’s *post hoc* test with significance defined as *p* < 0.05. Additionally, the area under the curve (AUC) was generated for gamma power and treatment effects were examined by separate ordinary one-way ANOVAs. Total activity counts were summed for time points 0–5 h post compound administration. A repeated measure one-way ANOVA was applied to examine the summation of activity. When significant, Dunnett’s *post hoc* tests were performed. In all cases significance was defined as *p* < 0.05.

## Results

### Polysomnography

#### M-5MPEP and VU0424238 Decrease REM Sleep Duration and Increase REM Sleep Latency

**FIGURE 1:**
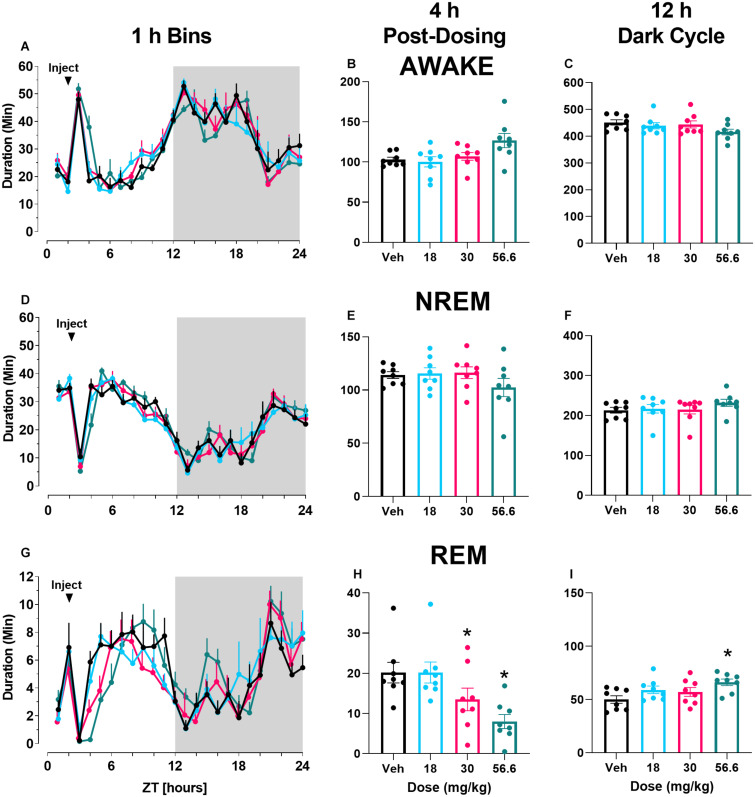
M-5MPEP dose-dependently reduced duration of time in REM sleep. Time spent in wake **(A–C)**, NREM **(D–F)**, and REM **(G–I)** sleep in 1 h bins **(A,D,G)** over a 24 h time period, summed duration over the first 4 h after M-5MPEP administration **(B,E,H)** or the summed duration over the 12 h dark period **(C,F,I)**. M-5MPEP was administered 2 h into the light period, noted by arrow. *p < 0.05 compared to respective time in the vehicle-treated condition. Line and bar graphs represent mean ± SEM (*n* = 8); individual circles on bar graphs depict individual data points. Gray rectangle around ZT 12–24 represents dark period.

The mGlu_5_ partial NAM M-5MPEP did not affect wake durations when examined in 1 h bins across a 24 h period. There was a main effect of time of day but no main effect of dose nor a significant interaction (Figure 1A; for statistics, see Table 1). To examine subtle effects accumulating over a longer duration, the sum of time in each stage was evaluated for the 4 h period following compound administration. There was a main effect of M-5MPEP dose on duration of time spent awake in the 4 h period following administration, but none were significantly different from vehicle (Figure 1B; for statistics, see Table 1). Additionally, we examined the summed duration during the subsequent 12 h active phase (dark cycle) that followed administration to look at potential rebound effects. M-5MPEP did not affect time spent awake during the 12 h dark cycle (Figures 1C; for statistics, see Table 1). When examining NREM sleep in 1 h bins across the 24 h period, M-5MPEP had a main effect of time of day but no main effect of dose or interaction (Figure 1D; for statistics, see Table 1). Similarly, M-5MPEP produced no significant alterations in NREM sleep during the 4 h period following compound administration and the 12 h dark cycle (Figures 1E,F; for statistics, see Table 1). Lastly, when assessing REM sleep durations, there was a main effect of time of day but no main effect of dose or interaction following M-5MPEP administration when assessed in 1 h bins across the 24 h period (Figure 1G; for statistics, see Table 1). Interestingly, there was a main effect of M-5MPEP on REM sleep duration during the 4 h period following administration (Figure 1H). *Post hoc* analysis revealed significant decreases in REM sleep following administration of the 30 and 56.6 mg/kg dose of M-5MPEP (for statistics, see Table 1). Lastly, there was also a main effect of M-5MPEP dose on REM sleep during the 12 h dark cycle (Figure 1I). *Post hoc* analysis revealed a significant increase in time spent in REM only at the 56.6 mg/kg dose of M-5MPEP (for statistics, see Table 1).

**TABLE 1:** Statistics for Figures 1–3.

Measure	Comparison	DF	*F*	*P*	*	Figure	*Post hoc* results	Significant time Points (hr)
M-5MPEP								
Wake (1 h bins)	Dose	2.20, 15.41	0.467	0.6534	ns			
	Time	4.06, 28.44	33.68	<0.0001	****	1A		
	Dose × Time	5.17, 36.16	0.964	0.4212	ns			
NREM (1 h bins)	Dose	2.55, 17.87	0.677	0.555	ns			
	Time	4.30, 30.08	35.18	<0.0001	****	1D		
	Dose × Time	5.28, 36.99	1.022	0.3105	ns			
REM (1 h bins)	Dose	2.21, 15.45	1.164	0.3427	ns			
	Time	4.38, 30.68	13.92	<0.0001	****	1G		
	Dose × Time	5.72, 40.07	1.233	0.3105	ns			
Wake (4 h Sum)	Dose	2.18, 15.24	3.911	0.0397	*	1B	ns	
NREM (4 h Sum)	Dose	2.22, 15.56	1.861	0.1863	ns	1E		
REM (4 h Sum)	Dose	1.83, 12.83	8.673	0.0048	**	1H	30 and 56.6 mg/kg	
Wake (dark cycle)	Dose	1.97, 13.75	3.737	0.0512 06	ns	1C		
NREM (dark cycle)	Dose	2.06, 14.40	1.978	0.1735	ns	1F		
REM (dark cycle)	Dose	1.59, 11.11	4.5	0.0436	*	1I	56.6 mg/kg	
Latency to REM	Dose	2.22, 15.54	6.499	0.0076	**	3A	56.6 mg/kg	
Latency to NREM	Dose	2.36, 16.50	0.473	0.662	ns	3B		
VU0424238								
Wake (1 h bins)	Dose	1.67, 11.72	4.104	0.0502	ns		Vehicle v 3 mg/kg	4, 8
	Time	4.62, 32.33	23.02	<0.0001	****	2A	Vehicle v 10 mg/kg	4, 5, 15
	Dose × Time	6.24, 43.67	2.412	0.0404	*		Vehicle v 30 mg/kg	4, 5, 20
NREM (1 h bins)	Dose	1.67, 11.72	9.824	0.0042	**		Vehicle v 3 mg/kg	4, 6–8
	Time	4.40, 30.82	33.42	<0.0001	****	2D	Vehicle v 10 mg/kg	4, 7, 8
	Dose × Time	6.35, 44.42	2.215	0.0561	ns		Vehicle v 30 mg/kg	4, 5, 20
REM (1 h bins)	Dose	2.55, 17.82	5.596	0.009	**		Vehicle v 3 mg/kg	4–7, 15
	Time	4.89, 34.23	10.06	<0.0001	****	2G	Vehicle v 10 mg/kg	4–8, 10, 15
	Dose × Time	5.74, 40.15	3.885	0.0042	**		Vehicle v 30 mg/kg	4–10, 18, 20
Wake (4 h sum)	Dose	2.22, 14.89	11.3	<0.0001	****	2B	3, 10, and 30 mg/kg	
NREM (4 h sum)	Dose	2.17, 15.17	9.65	0.0017	**	2E	10 and 30 mg/kg	
REM (4 h sum)	Dose	2.27, 15.86	125	<0.0001	****	2H	3, 10, and 30 mg/kg	
Wake (dark cycle)	Dose	2.09, 14.62	6.182	0.0107	*	2C	30 mg/kg	
NREM (dark cycle)	Dose	2.225, 15.57	5.873	0.0108	*	2F	30 mg/kg	
REM (dark cycle)	Dose	2.16, 15.09	2.56	0.1074	ns	2I	30 mg/kg	
Latency to REM	Dose	1.55, 10.83	16.58	0.0008	***	3C	3, 10, and 30 mg/kg	
Latency to NREM	Dose	1.62, 11.31	0.853	0.4289	ns	3D		

**FIGURE 2:**
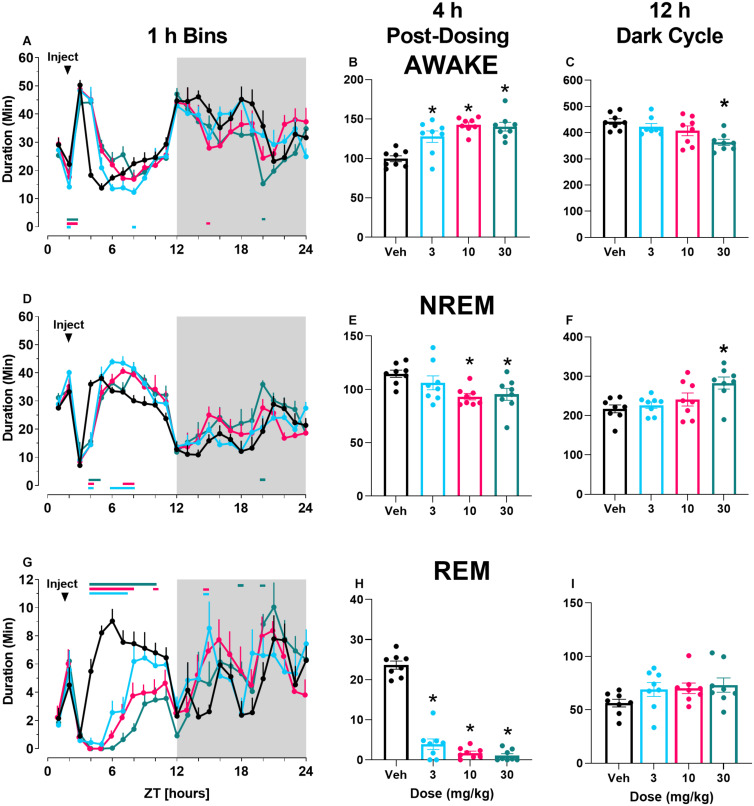
VU0424238 increased time awake and decreased sleep time. Time spent in wake **(A–C)**, NREM **(D–F)**, and REM **(G–I)** sleep in 1 h bins **(A,D,G)** over a 24 h time period, summed duration over the first 4 h after VU0424238 administration **(B,E,H)** or the 12 h duration summed over the 12 h dark period **(C,F,I)**. VU0424238 was administered 2 h into the light period, noted by arrow. Corresponding horizontal colored lines **(A,D,G)** represent time points at which VU0424238 doses were statistically different from respective time in the vehicle-treated condition (*p* < 0.05); *p < 0.05 compared to vehicle-treated condition. Line and bar graphs represent mean ± SEM (*n* = 8); individual circles on bar graphs depict individual data points. Gray rectangle around ZT 12–24 represents dark period.

In contrast to M-5MPEP, there was a main effect of time of day and a significant interaction following VU0424238 administration on time spent awake when examined in 1 h bins across the 24 h period (Figure 2A; for statistics, see Table 1). VU0424238 produced significant, dose-dependent increases in wake during the light cycle followed by significant reductions in wake in the dark cycle at the 10 mg/kg and 30 mg/kg doses. Additionally, during the 4 h following administration, there was a main effect of VU0424238 dose on time spent awake, and *post hoc* analysis revealed all tested doses produced significant increases in time awake when compared to the vehicle treatment (Figure 2B; for statistics, see Table 1). VU0424238 also produced decreases in time spent awake during the 12 h dark cycle (Figure 2C). *Post hoc* analyses revealed significance only at the 30 mg/kg dose of VU0424238 (for statistics, see Table 1). When examining NREM sleep in 1 h bins across the 24 h period, there was a main effect of dose and time-of-day following VU0424238 administration (Figure 2D). Dose-dependent alterations occurred in NREM sleep in the light cycle, and significant increases in NREM sleep occurred during the dark cycle at the 30 mg/kg dose (for statistics, see Table 1). There was also a main effect of VU0424238 dose on time spent in NREM during the 4 h period and the 12 h period (Figures 2E,F). *Post hoc* analyses indicated significant decreases in NREM sleep duration at the 10 and 30 mg/kg dose compared to vehicle treatment during the 4 h period followed by significant increases at 30 mg/kg during the 12 h dark cycle (for statistics, see Table 1). Lastly, there was a main effect of time of day and dose and a significant interaction following VU0424238 administration on REM sleep duration (Figure 2G). All doses produced significant decreases in REM sleep during the light cycle followed by increases during the dark cycle (for statistics, see Table 1). Additionally, there was a main effect of all tested doses of VU0424238 on duration of time spent in REM during the 4 h period, but no significance was found during the 12 h dark cycle (Figures 2H,I; for statistics, see Table 1).

**FIGURE 3:**
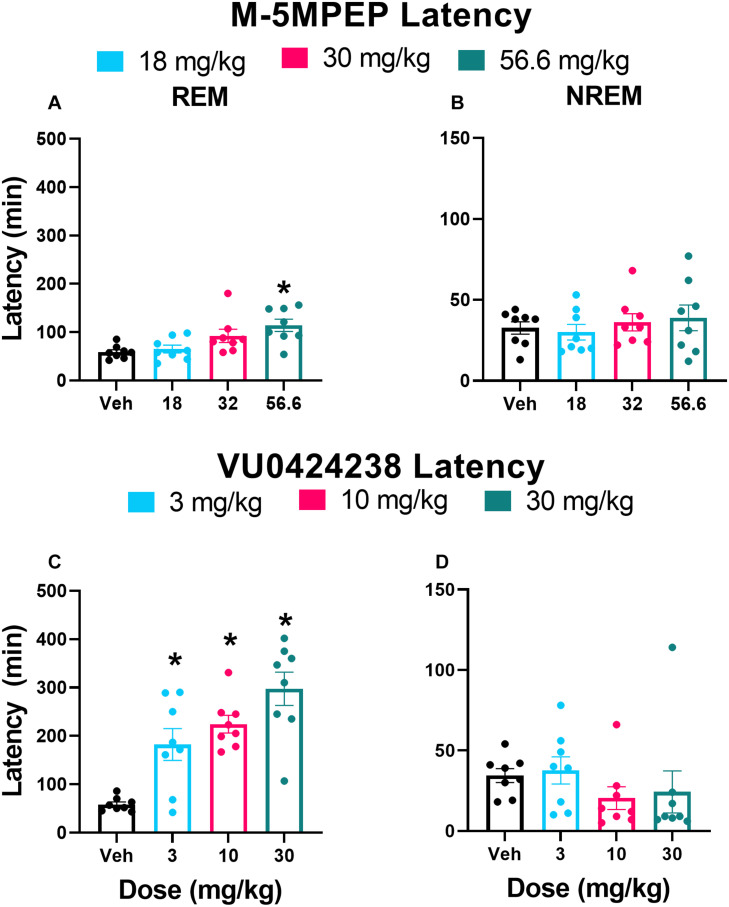
M-5MPEP and VU0424238 both increase latency to REM sleep. Latency to REM **(A,C)**, and NREM **(C,D)** sleep were assessed following administration of either M-5MPEP **(A,B)** or VU0424238 **(C,D)**. *p < 0.05 compared to respective vehicle-treated condition. Bars show mean ± SEM (*n* = 8); circles depict individual data points.

Finally, there was a main effect of both M-5MPEP and VU0424238 dose on latency to REM sleep (Figures 3A,C) but not latency to NREM sleep (Figures 3B,D). *Post hoc* analyses revealed significant increases in REM latency at the 56.6 mg/kg dose of M-5MPEP and all tested doses of VU0424238 when compared to the vehicle treatment (for statistics, see Table 1).

### Paired-Associates Learning Task

#### M-5MPEP and VU0424238 Alone Do Not Affect Accuracy

**FIGURE 4:**
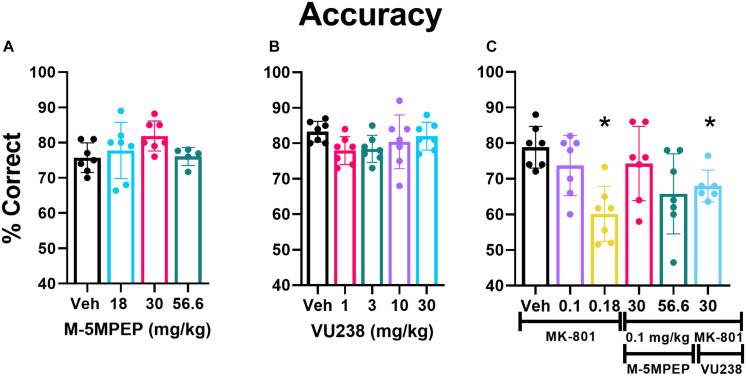
VU0424238 in combination with MK-801, but neither compound alone, disrupted accuracy on the PAL task. Bars depict group data as mean (±SEM) and circles within/above each bar depict individual data are shown for percent accuracy on the PAL task. M-5MPEP **(A)** and VU0424238 (VU238) **(B)** did not alter % accuracy on the PAL task. MK-801 alone dose-dependently decreased accuracy, and VU0424238 in combination with 0.1 mg/kg MK-801 **(C)** decreased accuracy. *p < 0.05 compared to vehicle-treated condition.

Of the 12 rats initially utilized for this study, 7 acquired the PAL task in an average of 56 sessions (average ± SEM 56.4 ± 5.3 sessions; range 38–82 sessions). Five failed to acquire the task following 90 training sessions and were excluded from the study. Statistical analysis for accuracy excluded two rats at the 56.6 mg/kg dose of M-5MPEP because <10 trials were completed on both test days, and only one of two sessions were included in the analysis for a separate two rats given they completed >10 trials only on the second test day of that dose. There was no main effect of dose for M-5MPEP on accuracy (*F*2.066,11.02 = 2.219, *p* = 0.3793) (Figure 4A) or on the percent of total trials that were CTs (*F*1.859,11.16 = 2.594, *p* = 0.1211) (Supplementary Figure 1A). There was a significant reduction in the number of selection trials completed (*F*1.271,7.623 = 18.94, *p* = 0.002). *Post hoc* analysis revealed a significant reduction in responding at the 56.6 mg/kg dose compared to vehicle treatment (Supplementary Figure 1D). When examining effects of VU0424238, there was no significant effect of dose found on accuracy (*F*1.885,11.31 = 1.853, *p* = 0.2020) (Figure 4B), percent of total trials that were CTs (*F*2.726,16.35 = 0.9584, *p* = 0.4286) (Supplementary Figure 1B), or number of selection trials completed (*F*1.023,7.673 = 2.251, *p* = 0.1737) (Supplementary Figure 1E).

#### VU0424238, but Not M-5MPEP, Decreased Accuracy in Combination With MK-801

There was a main effect of MK-801 dose found on accuracy (*F*2.838,16.46 = 9.048, *p* = 0.001) (Figure 4C) and percent of total trials that were CTs (*F*2.984,17.90 = 9.143, *p* < 0.001) (Supplementary Figure 1C). *Post hoc* comparisons revealed a significant reduction in accuracy and a significant increase in the percent of trials that were CTs at the 0.18 mg/kg dose relative to vehicle. There was also a main effect of MK-801 dose on the number of selection trials completed, but *post hoc* analyses revealed no significant differences (*F*2.128,12.77 = 4.415, *p* < 0.05; Supplementary Figure 1F).

0.1 mg/kg of MK-801 was selected to examine in combination with mGlu_5_ NAMs, since it did not significantly affect performance alone. Two doses of M-5MPEP, 30 and 56.6 mg/kg were examined in combination with MK-801 because these doses produced anxiolytic-like and antidepressant-like activity (Gould et al., 2015), selectively decreased REM sleep, and are associated with ∼80% receptor occupancy (Gould et al., 2015). The highest dose of VU0424238, 30 mg/kg, was chosen because it is associated with similar receptor occupancy and behavioral effects and is at the higher end of the dose-effect curve (Felts et al., 2017). A one-way ANOVA revealed a main effect of dose on accuracy (*F*2.838,16.46 = 9.048, *p* = 0.001) (Figure 3C). *Post hoc* comparisons revealed that the 30 mg/kg dose of VU0424238 combined with 0.1 mg/kg MK-801 significantly reduced accuracy compared to vehicle-treated rats. There was no significant effect of either M-5MPEP dose combination on accuracy. The group average in Figure 4C suggests that percent correct was similar between 56.6 mg/kg M-5MPEP and 30 mg/kg VU0424238 administered in combination with 0.1 mg/kg MK-801. However, individual values exhibit increased variability following administration of M-5MPEP with MK-801 and performance from one rat is driving the group average down. There was also a significant effect of dose on percent of trials that were CTs (*F*2.984,17.90 = 9.143, *p* < 0.001) (Supplementary Figure 1C). *Post hoc* comparisons showed a significant effect of only 30 mg/kg VU0424238 combined with MK-801. Lastly there was a main effect of treatment on number of selection trials completed, but *post hoc* analyses revealed no significance at any specific dose (*F*2.128,12.77 = 4.415, *p* < 0.05) (Supplementary Figure 1F).

### Quantitative Electroencephalography

#### VU0424238, but Not M-5MPEP, Produced Dose-Dependent Increases in Gamma Power

**FIGURE 5:**
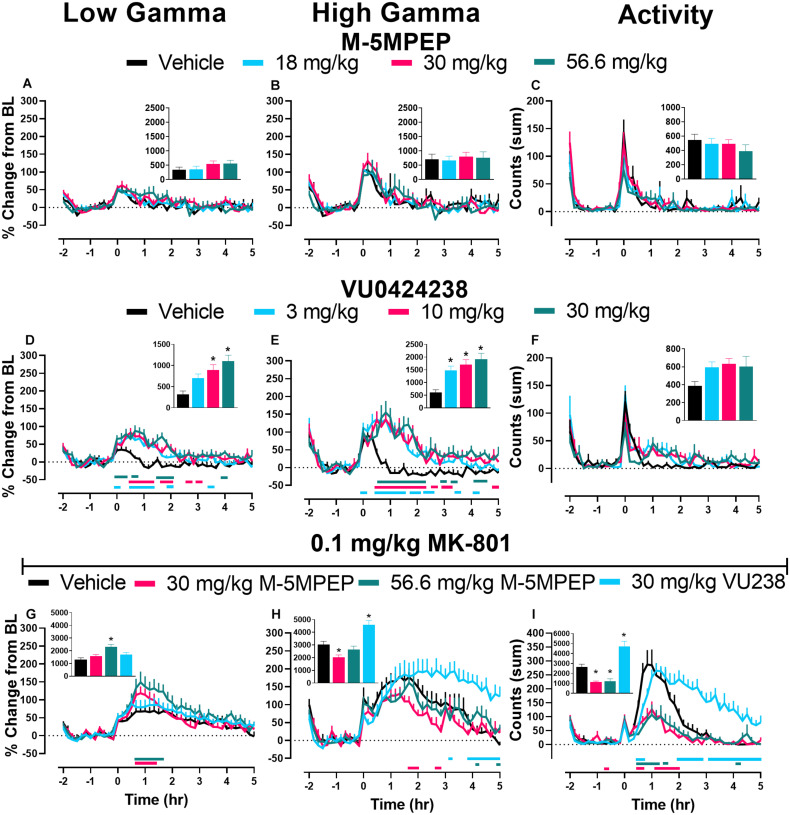
VU0424238, but not M-5MPEP, increased gamma power and potentiated MK-801-induced elevations. Data shown are group means ± SEM (*n* = 8) presented in 10 min bins for effects of M-5MPEP **(A–C)**, VU0424238 **(D–F),** and combinations with 0.1 mg/kg MK-801 **(G–I)** on low **(A,D,G)** and high frequency **(B,E,H)** gamma power and activity **(C,F,I)**. qEEG gamma power is expressed as a percent change from the average of each individuals’ 90-min baseline just prior to compound administration. Activity is expressed as summed counts in 10 min bins. Insets represent area under the curve (AUC) from time points 0–5 h post administration **(A,B,D,E,G,H)** or sum of all activity **(C,F,I)**. M-5MPEP and VU0424238 were administered at time point 0; Vehicle or MK-801 were administered 30 minutes after administration of mGlu_5_ NAMs. Time point -2 corresponds to ZT 0 and time point 5 corresponds to ZT 7. Horizontal colored lines represent time points at which treatment groups were statistically different from respective timepoints of the vehicle-treated group. *p < 0.05 compared to vehicle-treated rats.

In most frequency bands, a transient change in power was present and dissipated following vehicle administration within ∼30 min, coinciding with animal handling and ip injections (see Figure 5 and Supplementary Figures 2, 3). M-5MPEP did not affect low or high gamma power when compared to vehicle administration (Figures 5A,B). There was a main effect of dose and time following treatment with VU0424238 on both low and high gamma power during wake and a significant interaction of dose and time on high gamma power (see Figures 5D,E respectively; for all statistics see Table 2). *Post hoc* analysis revealed increases in low and high gamma following administration of 3, 10, and 30 mg/kg VU0424238 compared to the vehicle-treated condition at various 10 min time points within the 5 h following drug administration (see Table 2 for specific time points and colored horizontal lines on Figures 5D,E). Statistical analyses of area under the curve (AUC) from time 0–5 h after compound administration also revealed that 10 and 30 mg/kg VU0424238 maintained elevations in low frequency gamma power and 3, 10, and 30 mg/kg VU0424238 maintained elevations in high frequency gamma power relative to vehicle (Figures 5D,E insets; for statistics see Table 2). Spectral power frequencies below 30 Hz are shown in Supplementary Figures 2, 3 and statistics are presented in Supplementary Table 1. There were significant changes in theta, alpha, sigma, and beta bands following VU0424238 administration (Supplementary Figures 3A–F and Supplementary Table 1). Following administration of M-5MPEP, there was a main effect of time but not dose or time × dose interaction on percent change from baseline across theta, alpha, sigma, beta, low and high gamma frequency power bands during wake (see Figures 5A,B and Supplementary Figures 2A–F; for all statistics, see Table 2 and Supplementary Table 1). Briefly, there was a main effect of time but not dose on percent change from baseline in the delta band for both compounds. There were main effects of time of day on activity counts following both M-5MPEP and VU0424238 administration but no main effects of dose or time × dose interactions (Supplementary Figures 2, 3). Further examination also revealed no significant effect of either compound on total activity counts when summed over the 5 h recording period (see Figures 5C,F; for statistics see Table 2).

**TABLE 2:** Statistics for Figure 5.

Measure	Comparison	DF	*F*	*P*	*	Figure	*Post hoc* results	Significant Time Points (min)
M-5MPEP								
Low gamma	Dose	2.29, 16.05	0.581	0.7643	ns			
	Time	5.07, 35.47	9.488	<0.0001	****	5A		
	Dose × Time	6.01, 41.26	1.014	0.4297	ns			
	AUC	3, 28	1.258	0.3079	ns			
High gamma	Dose	2.31, 16.18	0.201	0.8485	ns			
	Time	4.05, 28.31	15.9	<0.0001	****	5B		
	Dose × Time	5.65, 38.84	0.774	0.5886	ns			
	AUC	3, 28	0.106	0.9557	ns			
Activity	Dose	1.58, 11.03	3.388	0.0793	ns			
	Time	2.85, 19.92	22.16	<0.0001	****	5C		
	Dose × Time	5.14, 35.35	1.3	0.2858	ns			
	5 h sum	2.38, 16.63	1.953	0.1682	ns			
VU0424238								
Low gamma	Dose	1.97, 13.80	8.87	0.0034	**		Veh v 3 mg/kg	0, 30–80, 120, 210
	Time	4.53, 31.69	14.21	<0.0001	****	5D	Veh v 10 mg/kg	30–80, 100–120, 160, 180
	Dose × Time	5.89, 40.26	2.16	0.0688	ns		Veh v 30 mg/kg	0–20, 40–120, 240
	AUC	3, 28	8.16	0.0005	***		10 and 30 mg/kg	
High gamma	Dose	1.80, 12.58	17.24	0.0006	***		Vehicle v 3 mg/kg	0, 30–90, 110, 120, 140, 150, 210, 250
	Time	3.87, 27.05	14.73	<0.0001	****	5E	Vehicle v 10 mg/kg	30–130, 160, 180, 190, 300
	Dose × Time	5.37, 36.70	2.87	0.0251	*		Vehicle v 30 mg/kg	40–130, 180, 200, 250–270
	AUC	3, 28	9.779	0.0001	***		3, 10, and 30 mg/kg	
Activity	Dose	1.53, 10.68	3.083	0.0966	ns			
	Time	3.98, 27.86	15.44	<0.0001	****	5F		
	Dose × Time	5.50, 37.57	1.87	0.1141	ns			
	5 h Sum	1.24, 8.71	4.26	0.0645	ns			
Combinations with 0.1 mg/kg MK-801								
Low gamma	Dose	1.89, 13.24	3.279	0.0718	ns		Veh v 30 mg/kg M-5MPEP	40–80
	Time	1.76, 12.33	22.77	0.0001	***	5G	Veh v 56.6 mg/kg M-5MPEP	40–100
	Dose × Time	5.78, 39.93	2.587	0.0421	*			
	AUC	3, 28	7.087	0.0011	**		56.6 mg/kg M-5MPEP	
High gamma	Dose	1.27, 8.92	5.711	0.0351	*		Veh v 30 mg/kg M-5MPEP	100–120, 160, 170
	Time	2.79, 19.56	24.27	<0.0001	****	5H	Veh v 56.6 mg/kg M-5MPEP	250, 300
	Dose × Time	4.50, 31.03	5.444	0.0014	**		Veh v 30 mg/kg VU0424238	190, 230–300
	AUC	3, 28	16.82	<0.0001	****			
Activity	Dose	1.62, 11.34	34.85	<0.0001	****		Veh v 30 mg/kg M-5MPEP	–40, 30, 40, 70–120
	Time	3.02, 21.12	26.31	<0.0001	****	5I	Veh v 56.6 mg/kg M-5MPEP	30–70, 90, 250
	Dose × Time	4.67, 32.22	8.346	<0.0001	****		Veh v 30 mg/kg VU0424238	30, 40, 120–170, 190–300
	5 h Sum	1.70, 11.87	40.95	<0.0001	****		30 and 56.6 mg/kg M-5MPEP 30 mg/kg VU0424238	

Delta power during NREM sleep was also assessed as a measure of sleep quality (Steiger and Kimura, 2010; Gould et al., 2016). There was only a main effect of time following M-5MPEP administration (Supplementary Figure 5A; for statistics, see Supplementary Table 1). There was a main effect of dose, time, and a time × dose interaction following VU0424238 administration. *Post hoc* analyses revealed that VU0424238, but not M-5MPEP, dose-dependently increased delta power compared to vehicle treatment from 2–4 h (3 mg/kg), 2–8 h (10 mg/kg), and 2–14 h (30 mg/kg) following administration (Supplementary Figure 5B; for statistics, see Supplementary Table 1).

#### M-5MPEP and VU0424238 in Combination With MK-801 Differentially Affected Gamma Power

We specifically focused on gamma power as previous studies reported that MK-801 and other NMDAR antagonists increase gamma power and because gamma power has correlated with psychotic-like symptoms in healthy humans (Javitt, 2007; Coyle et al., 2012; Phillips et al., 2012; Hiyoshi et al., 2014; Sanacora et al., 2014; Gould et al., 2016). Thus, this may serve as a translational biomarker for dose selection in human studies. Doses of mGlu_5_ NAMs administered in combination with 0.1 mg/kg MK-801 were selected to provide a functional readout for the effects on cognition in the PAL task. There was a main effect of time and a significant time × mGlu_5_ NAM treatment interaction on MK-801-induced effects on low gamma power. Dunnett’s *post hoc* analysis revealed significant increases in percent change from baseline following administration of the 30 and 56.6 mg/kg doses of M-5MPEP relative to the vehicle condition (vehicle + 0.1 mg/kg MK-801) at various time points within 5 h following drug administration (Figure 5G; for statistics, see Table 2). Statistical analysis of AUC from 0 to 5 h following administration showed a significant increase compared to MK-801 treatment alone at the 56.6 mg/kg dose of M-5MPEP (Figure 5G, inset; for statistics, see Table 2). There was also a main effect of time and time × dose interaction on high gamma power. *Post hoc* analyses revealed significant differences across all mGlu_5_ treatment combinations. Significant increases in high gamma power were found at only one 10-min bin for combination with 56.6 mg/kg of M-5MPEP but multiple bins for combination with 30 mg/kg VU0424238. Interestingly, combinations with 30 mg/kg of M-5MPEP produced significant reductions in high gamma power across multiple time points (see colored horizontal lines below Figure 5G; for statistics, see Table 2). AUC analysis showed a significant reduction relative to the vehicle condition following administration of 30 mg/kg M-5MPEP and a significant increase in AUC following administration of 30 mg/kg VU0424238 (Figure 5H; for statistics, see Table 2). Spectral power frequencies below 30 Hz are shown in Supplementary Figures 5A–E with statistics reported in Supplementary Table 1. Additionally, the average spectral power across the 5 h following compound administration in 1 Hz bins is shown in Supplementary Figure 6. Lastly, MK-801 alone increased activity counts consistent with prior reports (e.g., Andiné et al., 1999). There was a main effect of treatment, time and treatment × time interaction on activity counts. *Post hoc* analyses showed a significant difference in all three dose combinations compared to MK-801 administration alone, such that 30 and 56.6 mg/kg M-5MPEP decreased activity counts and 30 mg/kg VU0424238 increased activity counts at multiple time points across the 5 h period (see Figure 5I, see Table 2 for statistics). There was also a main effect of dose when analyzing the summed activity counts across the 5 h period with similar decreases and increases following administration of M-5MPEP and VU0424238 respectively (see Figure 5I, inset; for statistics, see Table 2).

## Discussion

Functional inhibition of mGlu_5_ represents a promising treatment approach for multiple disorders including depression, anxiety, substance use disorder and comorbid sleep disruptions, yet mitigating the risk of adverse effects remains a concern for clinical development (McGeehan and Olive, 2003; Busse et al., 2004; Dölen et al., 2007; Morin et al., 2010; Lindemann et al., 2015; Gould et al., 2018). The partial and full mGlu_5_ NAMs M-5MPEP and VU0424238, respectively, decreased REM sleep duration and increased latency to REM sleep, extending knowledge of the antidepressant-like effects of these novel mGlu_5_ NAMs. Importantly, both compounds were devoid of cognitive and functional indicators of adverse effects within dose ranges that correspond with antidepressant- and/or anxiolytic-like effects in rodents (Gould et al., 2015; Felts et al., 2017). In contrast with VU0424238, M-5MPEP did not affect working memory or potentiate high gamma power when administered in combination with MK-801. Together, using translational measures of behavior and brain function, these data support the hypothesis that NMDAR inhibition may underlie some of the adverse effects associated with inhibition of mGlu_5_. Furthermore, submaximal functional antagonism of mGlu_5_ via partial mGlu_5_ NAMs represents a viable approach to pursue therapeutic effects for multiple CNS disorders while minimizing adverse effects.

Polysomnography studies extended our understanding of the dose-dependent effects of the partial mGlu_5_ NAM M-5MPEP and suggest it may have promising antidepressant-like alterations on sleep. Sleep disturbances are prevalent yet underappreciated symptoms associated with most psychiatric disorders, and polysomnography has gained recent support as a translational biomarker for screening possible antidepressant-like effects (Wichniak et al., 2013; Lindemann et al., 2015). Aberrant increases in REM sleep and a shorter REM sleep latency are common symptoms of patients diagnosed with MDD (for review see Steiger and Pawlowski, 2019). In general, current antidepressant medications suppress REM sleep and increase REM sleep latency without concomitant decreases in NREM sleep duration or latencies (for review see Wichniak et al., 2013, 2017; Steiger and Pawlowski, 2019). M-5MPEP displayed a similar profile to antidepressant medications, producing selective, dose-dependent decreases in REM sleep duration and increases in latency without altering NREM sleep. At the highest tested dose of M-5MPEP, there was a rebound effect in REM sleep during the 12 h dark cycle. However, a rebound effect is expected in these healthy rats as it is a natural physiological response to maintain sleep homeostasis (Feriante and Singh, 2020). In contrast, VU0424238 demonstrated wake-promoting effects which were followed by non-selective decreases in NREM and REM sleep duration and increases in REM sleep latency, consistent with effects of first generation and recent full mGlu_5_ NAMs including MPEP, MTEP and basimglurant (Cavas et al., 2013; Harvey et al., 2013; Ahnaou et al., 2015; Lindemann et al., 2015). Importantly, VU0424238-induced increases in REM sleep latency were not influenced by an increase in NREM sleep latency because latency to first REM sleep bout following the first NREM sleep bout was still increased in a dose-dependent pattern (data not shown). We also examined delta power during NREM sleep as a biomarker of sleep quality (Steiger and Kimura, 2010). Many patients with MDD exhibit reduced delta power during NREM sleep, and some antidepressant medications have been shown to increase delta power during NREM sleep (Kupfer et al., 1989; Quera-Salva et al., 2010; Steiger and Kimura, 2010). Interestingly, delta power during NREM sleep (but not during time awake) was elevated following administration of VU0424238 but not by M-5MPEP (Supplementary Figures 2, 3, 5). NREM sleep duration during the subsequent 12 h dark cycle was increased following VU0424238 but not M-5MPEP, likely reflecting the NREM sleep rebound following acute wake-promoting effects (Franken et al., 1991; Oonk et al., 2016; Dispersyn et al., 2017). While insomnia has been reported in a majority of patients with MDD, hypersomnia is also reported in a smaller subset of patients (Armitage, 2007; Steiger and Pawlowski, 2019). While speculative, partial and full mGlu_5_ NAMs may both be efficacious depending on the sleep profile of the targeted subpopulation.

Present data also help mitigate concerns regarding cognitive deficits as a relevant adverse effect following functional antagonism of mGlu_5_. Although other mGlu_5_ NAMs have not yet been examined on this PAL task, effects of full mGlu_5_ NAMs on visuospatial memory function, as well as other cognitive domains, are equivocal and appear to be task- and dose- dependent (Simonyi et al., 2010). Cognitive impairments following systemic administration of MPEP or MTEP are reported at doses corresponding with >85% receptor occupancy, which is a higher dose range than needed to produce anxiolytic-like or antidepressant-like effects (for discussion, see Simonyi et al., 2010; Ahnaou et al., 2015) and may suggest off-target effects (Montana et al., 2009). Importantly, fenobam did not affect cognition in humans (Berry-Kravis et al., 2009; Cavallone et al., 2020). Moreover, full mGlu_5_ NAMs have even improved cognition in animal models of Fragile X (Michalon et al., 2014), Parkinson’s (De Leonibus et al., 2009), and Alzheimer’s Disease (Hamilton et al., 2016) reiterating mGlu_5_ function is relevant for cognition and effects on cognition (positive or negative) are also dependent on the underlying neurobiology of the disorder.

Consistent with our hypothesis, the full mGlu_5_ NAM VU0424238 but not the partial mGlu_5_ NAM M-5MPEP potentiated MK-801-induced disruptions on the PAL task, suggesting a functional readout for mGlu_5_-NMDAR interactions. NMDAR function is critical for synaptic plasticity changes underlying learning and memory function (Collingridge, 1987; Riedel et al., 2003; Robbins and Murphy, 2006) and is known to be augmented in a number of CNS disorders including depression, anxiety and schizophrenia (see Rush and Buisson, 2014; Maqsood and Stone, 2016). Pharmacological antagonism of NMDARs via PCP, ketamine, or MK-801 has negatively impacted cognitive performance in rodents and monkeys (Morris et al., 1986; Taffe et al., 2002; Day et al., 2003; Robbins and Murphy, 2006; Talpos et al., 2009, 2015; Neill et al., 2010). The PAL task has demonstrated high sensitivity to glutamatergic manipulation, including dose-dependent disruptions via MK-801 (Talpos et al., 2009, 2015). In contrast to M-5MPEP, when combined with a non-disruptive dose of MK-801, 30 mg/kg VU0424238 resulted in a significant reduction in accuracy as well as an increase in the number of correction trials. The significant increase in number of correction trials, similar to findings with higher doses of MK-801 alone, could be an indication of perseverative responding, another cognitive effect associated with CNS disorders related to NMDAR hypofunction (Bornstein et al., 1990; Szoke et al., 2008; Talpos et al., 2015). The present data add to prior behavioral studies showing that full mGlu_5_ NAMs including fenobam, MPEP, and MTEP potentiate the cognitive-impairing and hyperlocomotor effects of NMDAR antagonists PCP and MK-801 (Kinney et al., 2003; Homayoun et al., 2004; Gould et al., 2015).

Although hyperlocomotion is predominately used as a surrogate behavior for psychotomimetic-like effects in rodents following administration of NMDAR antagonists (Kinney et al., 2003; Homayoun et al., 2004), qEEG represents a more translational approach as a pharmacodynamic biomarker of brain function (Sanacora et al., 2014). Extensive research has shown that NMDAR antagonists, including MK-801, increase gamma frequency power, and excessive increases in gamma power are associated with both positive symptoms of schizophrenia and cognitive impairments in healthy humans and animals (Javitt, 2007; Coyle et al., 2012; Phillips et al., 2012; Hiyoshi et al., 2014; Gould et al., 2016). Consistent with previous literature, 0.1 mg/kg MK-801 increased gamma power. Interestingly, M-5MPEP decreased (30 mg/kg) or had no effect (56.6 mg/kg) on high gamma power but both doses significantly decreased MK-801-induced hyperactivity. Future studies will compare M-5MPEP with other partial mGlu_5_ NAMs to determine if this is relevant for any therapeutic outcome and/or if this is compound specific (Hiyoshi et al., 2014; Gould et al., 2016). In contrast to M-5MPEP, 30 mg/kg VU0424238 increased the duration of MK-801’s effects on gamma power and locomotion. Importantly, effects of VU0424238 on high gamma power persisted after activity decreased to near baseline levels. Cognitive testing was conducted during the dark (active) period when arousal and gamma power, one surrogate for arousal, are highest (e.g., Gould et al., 2016). MK-801 does not induce as robust of an increase in gamma power when administered in the dark period as compared to the light period (Supplementary Figures 7A–C, inset). The higher basal gamma power in the dark period likely contributed to an observed “ceiling effect”, and aberrant elevations in gamma power are not observed as extensively following administration of vehicle, M-5MPEP, and VU042438 with 0.1 mg/kg MK-801. Thus, the present study focused on changes in gamma power during the light cycle when basal gamma power is lower, allowing for a greater signal window to observe functional mGlu_5_-NMDAR interactions. Similar effects albeit with a lesser magnitude were present when the same interaction studies were conducted in the dark period (Supplementary Figure 6).

Both doses of M-5MPEP transiently potentiated MK-801-induced increases in low gamma power suggesting a functional interaction between M-5MPEP and NMDARs. Relative implications for selective alterations in low versus high frequency gamma band ranges are not completely understood from a biochemical or circuit perspective. Both gamma band ranges are implicated in working memory performance and psychosis, but high frequency gamma has further been implicated in visual and auditory processing (for review see Uhlhaas et al., 2011; Yadav et al., 2021). This may be more relevant for predicting psychotomimetic-like effects as deficits in these domains are commonly reported in schizophrenia. Importantly, potentiation of MK-801-induced effects on high gamma power corresponded with disruptions on cognitive function, whereas changes in low gamma power did not, suggesting that the high gamma frequency range may be more relevant for translating brain function with cognitive outcome for mGlu_5_-NMDAR interactions.

VU0424238, but not M-5MPEP, showed elevations in low and high gamma power as well as produced significant alterations in frequency bands between 5 and 30 Hz (Figure 4 and Supplementary Figures 2, 3) consistent with prior studies assessing MPEP and MTEP (Ahnaou et al., 2015). The significant and sustained elevations of VU0424238 on high gamma power may reflect increased arousal or wake-promoting effects. While the impact of VU0424238 on gamma power alone may have influenced elevations when administered in combination with MK-801, it is important to note potentiation of MK-801’s effects peaked and were sustained between hours 3 and 5 following administration whereas the effects of VU0424238 alone on high gamma power dwindled within this time range exhibiting multiple 10-min bins that were not significantly different from vehicle.

In summary, submaximal inhibition of mGlu_5_ is sufficient to engender multiple antidepressant-like behavioral effects including selective reduction of REM sleep without signs of adverse effects (i.e., deficits in working memory). However, present and previously reported antidepressant-like effects of M-5MPEP only occurred at doses corresponding with approximately 80% or greater *ex vivo* receptor occupancy (Rodriguez et al., 2005; Gould et al., 2015; Felts et al., 2017). In contrast, lower doses of VU0424238 (3 mg/kg) associated with <80% receptor occupancy exhibited similar decreases in REM sleep and anxiolytic-like effects in previous reports (Felts et al., 2017). This submaximal functional effect at high receptor occupancy by partial mGlu_5_ NAMs may influence the maximal therapeutic potential but also mitigate risk of adverse effects. VU0424238, but not M-5MPEP, disrupted cognition and altered brain function at doses associated with >80% mGlu_5_ receptor occupancy only when probed with a submaximal dose of MK-801. This pharmacological probe further supports mGlu_5_-NMDAR interactions. Present data may add to growing literature reducing concern for adverse effect liability surrounding newer, more selective full mGlu_5_ ligands (Montana et al., 2009). Future studies that use complex models of depression-like phenotypes as well as studies that investigate other cognitive domains are needed. However, given the heterogeneity of many neuropsychiatric disorders, individuals with underlying deficits in NMDAR function may still be at a greater risk for some adverse effects, and use of a partial mGlu_5_ NAM may be an advantageous treatment approach. The degree to which functional inhibition of mGlu_5_ would engender therapeutic and/or adverse effects in a heterogeneous population remains to be determined, but present data further support the use of translatable biomarkers such as qEEG to investigate this question in future animal and human studies.

## Data Availability Statement

The original contributions presented in the study are included in the article/Supplementary Material, further inquiries can be directed to the corresponding author/s.

## Ethics Statement

The animal study was reviewed and approved by Wake Forest University Animal Care and Use Committee.

## Author Contributions

KH: formal analysis, investigation, methodology, writing – original draft, and visualization. AL: formal analysis, investigation, and methodology. CML: investigation. EB and BP: formal analysis. LS: software. CWL and CJ: resources. RG: conceptualization, methodology, formal analysis, writing –reviewing and editing, visualization, and funding acquisition. All authors contributed to the article and approved the submitted version.

## Conflict of Interest

The authors declare that the research was conducted in the absence of any commercial or financial relationships that could be construed as a potential conflict of interest.
