# Supplementary material for: Partial mGlu5 Negative Allosteric Modulator M-5MPEP Demonstrates Antidepressant-Like Effects on Sleep Without Affecting Cognition or Quantitative EEG
Source: Front Neurosci. 2021 Jul 2;15:700822. doi: 10.3389/fnins.2021.700822 (PMC8283128; doi:10.3389/fnins.2021.700822)
Supplement: Supplementary file 1 [file Data_Sheet_1.PDF]

S1. Supplemental Methods

S1.1 Determining diurnal influences on inactive vs active phase gamma power. High gamma power during waking epochs in the active phase or the inactive phase following administration of vehicle, VU0424238, and M-5MPEP was evaluated to determine if effects of MK-801 and/or mGlu5 NAMs were influenced by diurnal rhythm. Custom MATLAB scripts averaged data by state (waking epochs only) in 10-min bins for 7 hours (2 h pre- and 5 h post-administration). The relationship between vehicle administered 2 h into the light cycle followed by administration of 0.1 mg/kg MK-801 30 min later and vehicle + administered 2 h into the dark cycle followed by administration of 0.1 mg/kg MK-801 30 min later was evaluated looking at within-session changes in relative power and when expressed as percent change from each individual's 90-min baseline directly prior to compound administration. Statistical analyses were performed by mixed effects two-way ANOVAs followed by Sidak's multiple comparison test with significance defined as $p<0.05$. Additionally, an unpaired t-test was used to compare relative power in the high gamma frequency range for the 2 h baseline. Lastly, within-session changes expressed as a percent change from each individual rat's 90-min baseline were observed for administration of vehicle, 56.6 mg/kg M-5MPEP, and 30 mg/kg VU0424238 administered 2 h into the dark cycle followed by administration of 0.1 mg/kg MK-801 30 min later. Statistical analyses were performed by mixed effects two-way ANOVAs followed by Dunnett's multiple comparison test with significance defined as $p<0.05$.

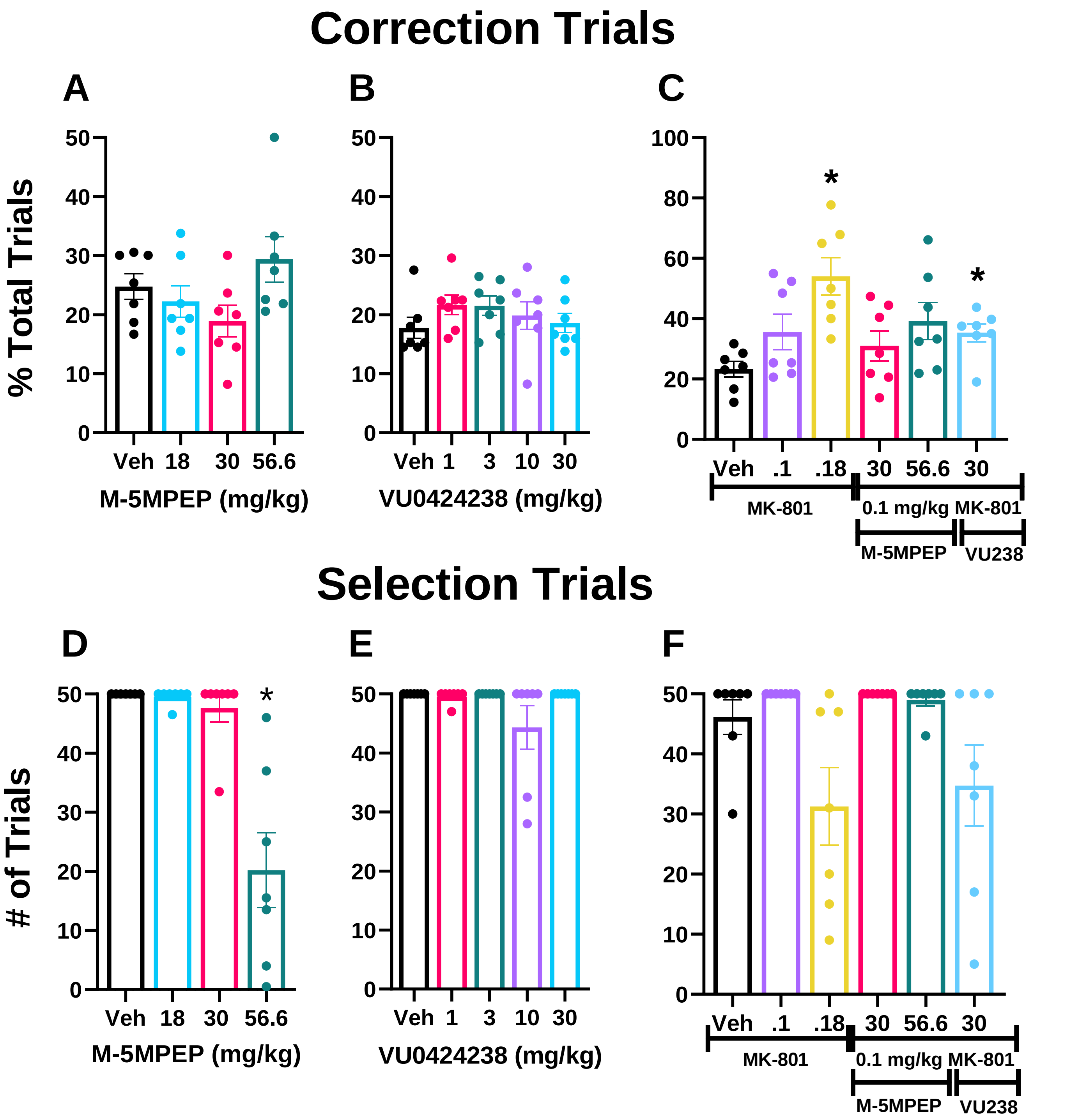

Figure 1. Effects of VU0424238 and M-5MPEP alone and in combination with MK-801 on number of correction and total trials completed. Correction trials are represented as the percentage of total trials (correction trials/[selection trials + correction trials] x 100). M-5MPEP **(A)** and VU0424238 **(B)** did not alter correction trials. MK-801 alone dose-dependently increased number of correction trials, and VU0424238 increased correction trials when in combination with MK-801 **(C)**. 56.6 mg/kg M-5PEP decreased number of selection trials **(D)** whereas there was no effect of VU0424238 alone **(E)** or MK-801 alone or in combination with mGlu$_5$ NAMs **(F)**. Individual data are represented by circles and mean ±SEM are shown as overlapping bar graphs. *p<0.05 compared to vehicle-treated mean.

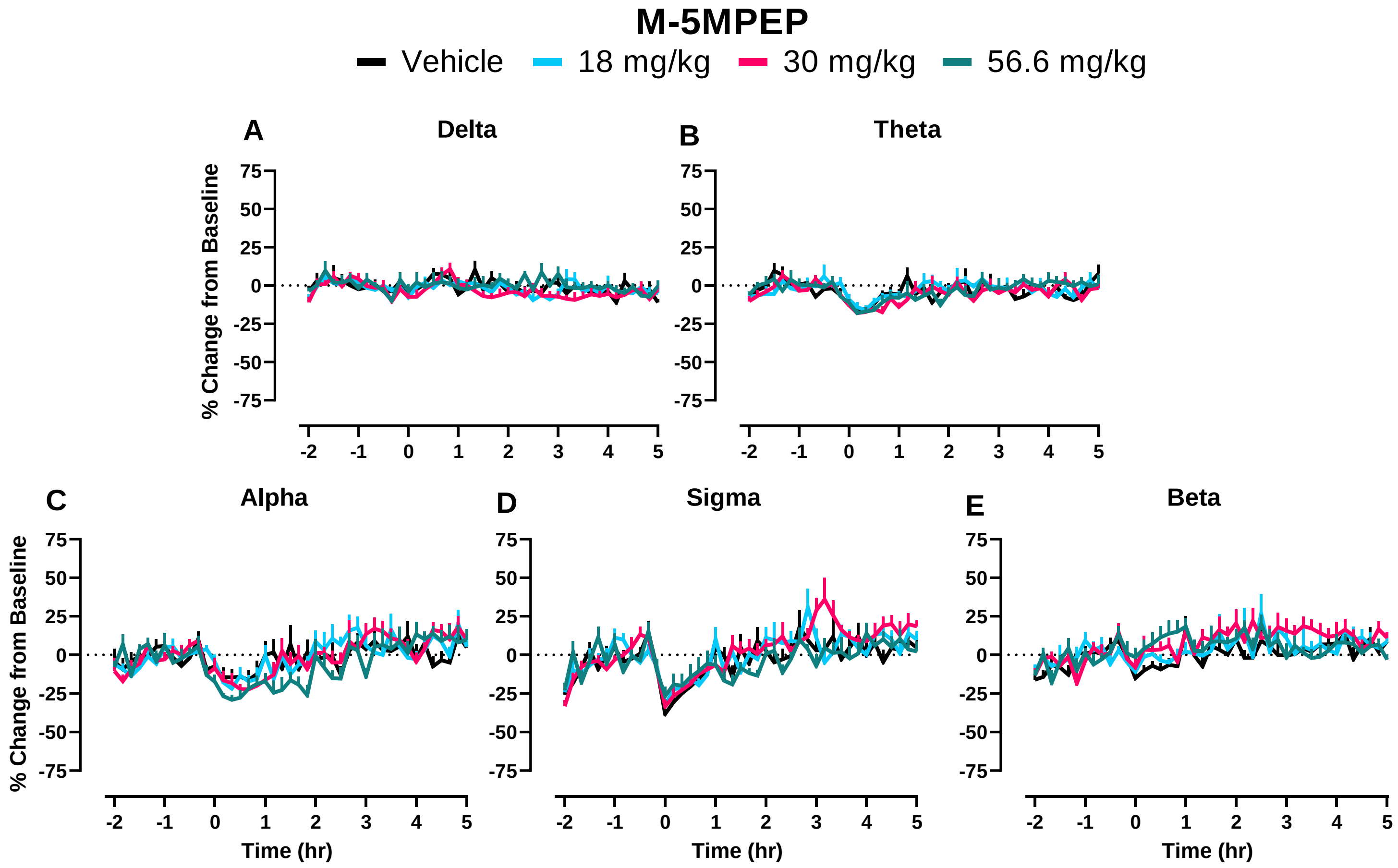

Figure 2. M-5MPEP had no effect on spectral power frequency bands below 30 Hz. Data shown are group means ±SEM (n=8) presented in 10 minutes bins for effects of M-5MPEP on delta **(A)** theta **(B)** alpha **(C)** sigma **(D)** and beta **(E)**. qEEG spectral power is expressed as a percent change from the average of a 90-minute baseline just prior to compound administration. M-5MPEP was administered at time point 0. Time point -2 corresponds to ZT 0 and time point 5 corresponds to ZT 7.

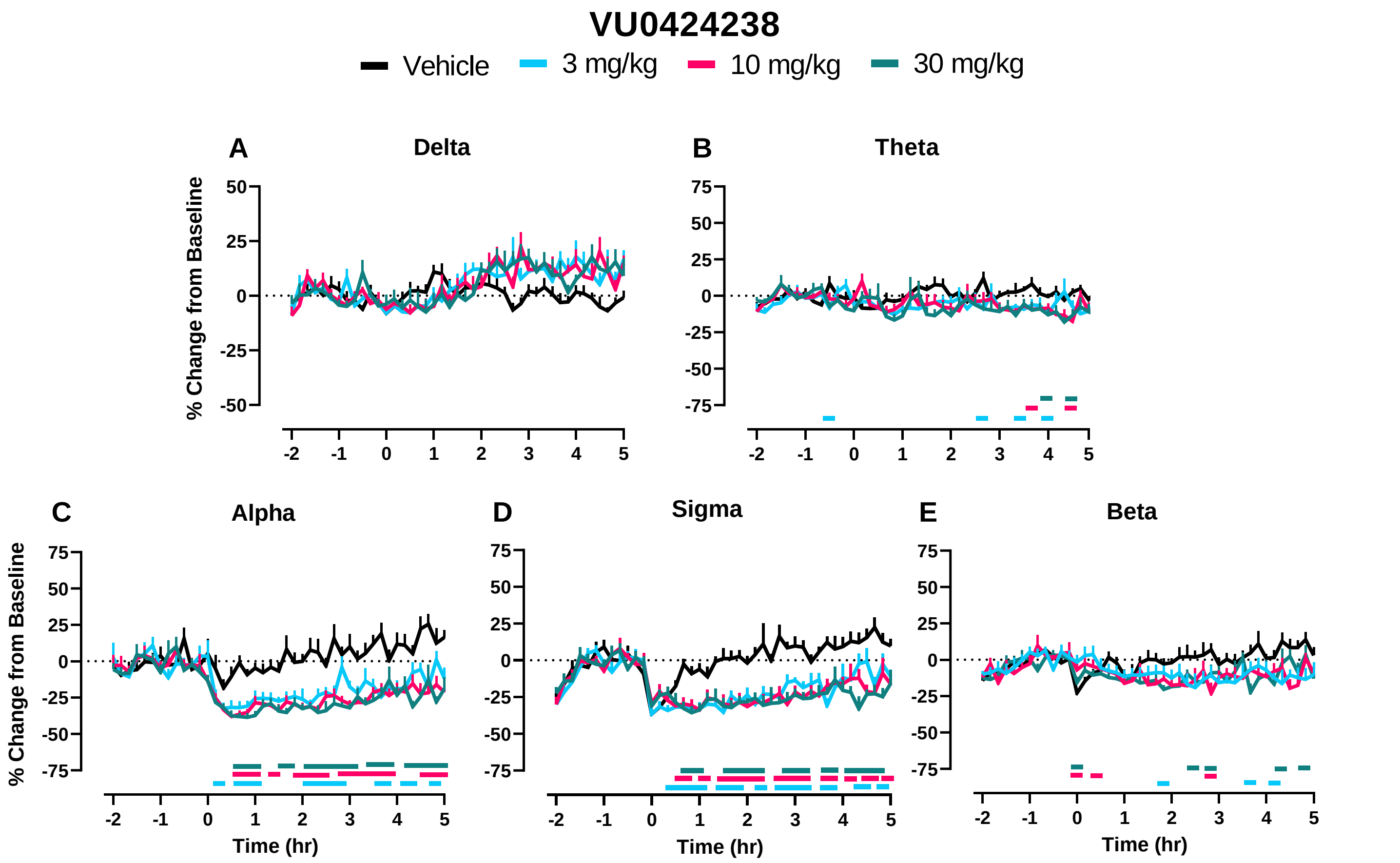

Figure 3. VU0424238 decreased alpha, sigma and beta power. Data shown are group means ±SEM (n=8) presented in 10 minutes bins for effects of VU0424238 on delta **(A)** theta **(B)** alpha **(C)** sigma **(D)** and beta **(E)**. qEEG spectral power is expressed as a percent change from the average of a 90-minute baseline just prior to compound administration. VU0424238 was administered at time point 0. Time point -2 corresponds to ZT 0 and time point 5 corresponds to ZT 7.

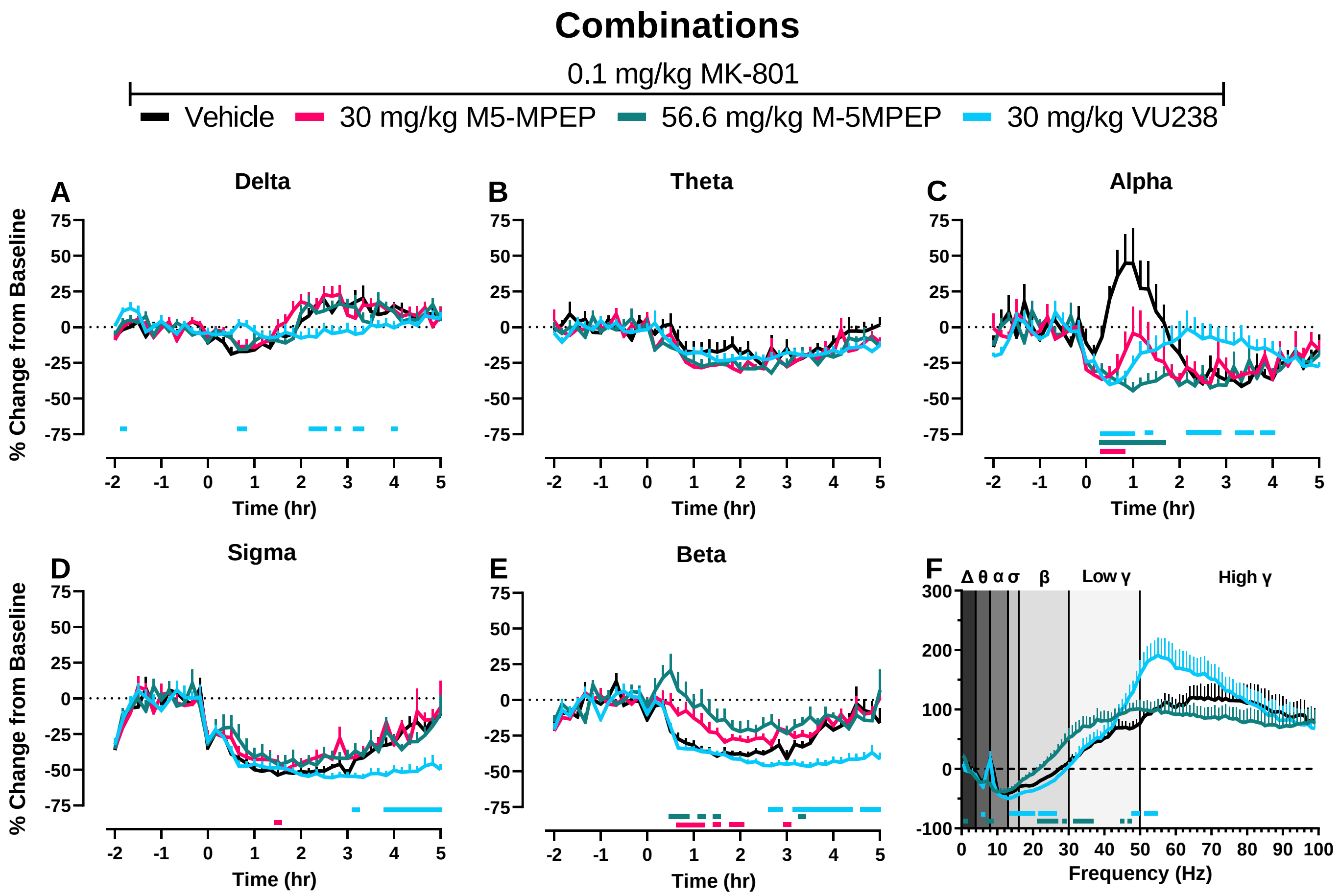

Figure 4. VU0424238 and M-5MPEP had differential effects on spectral power frequencies below 30 Hz when combined with MK-801. Data shown are group means ±SEM (n=8) presented in 10 minutes bins for effects of combinations with 0.1 mg/kg MK801 on **(A)** delta **(B)** theta **(C)** alpha **(D)** sigma and **(E)** beta. Vehicle, M-5MPEP, and VU0424238 were administered at time point 0; MK-801 was administered 30 minutes after administration of $mGlu_5$ NAMs. **(F)** Average power during wake epochs only over the 5 h post-dosing is shown as group mean ±SEM (n=8) in 1 Hz (0.5-100 Hz) bins. qEEG spectral power is expressed as a percent change from each individual's 90 min baseline prior to compound administration. Gray vertical bars represent frequency bands (delta 0.5-4 Hz, theta 4-8 Hz, alpha 8-13 Hz, sigma 13-15 Hz, beta 13-30 Hz, low gamma 30-50 Hz, high gamma 50-100 Hz). Corresponding colored horizontal lines represent time points **(A-E)** or frequencies **(F)** at which treatment groups were statistically different from the vehicle + 0.1 mg/kg group, p<0.05

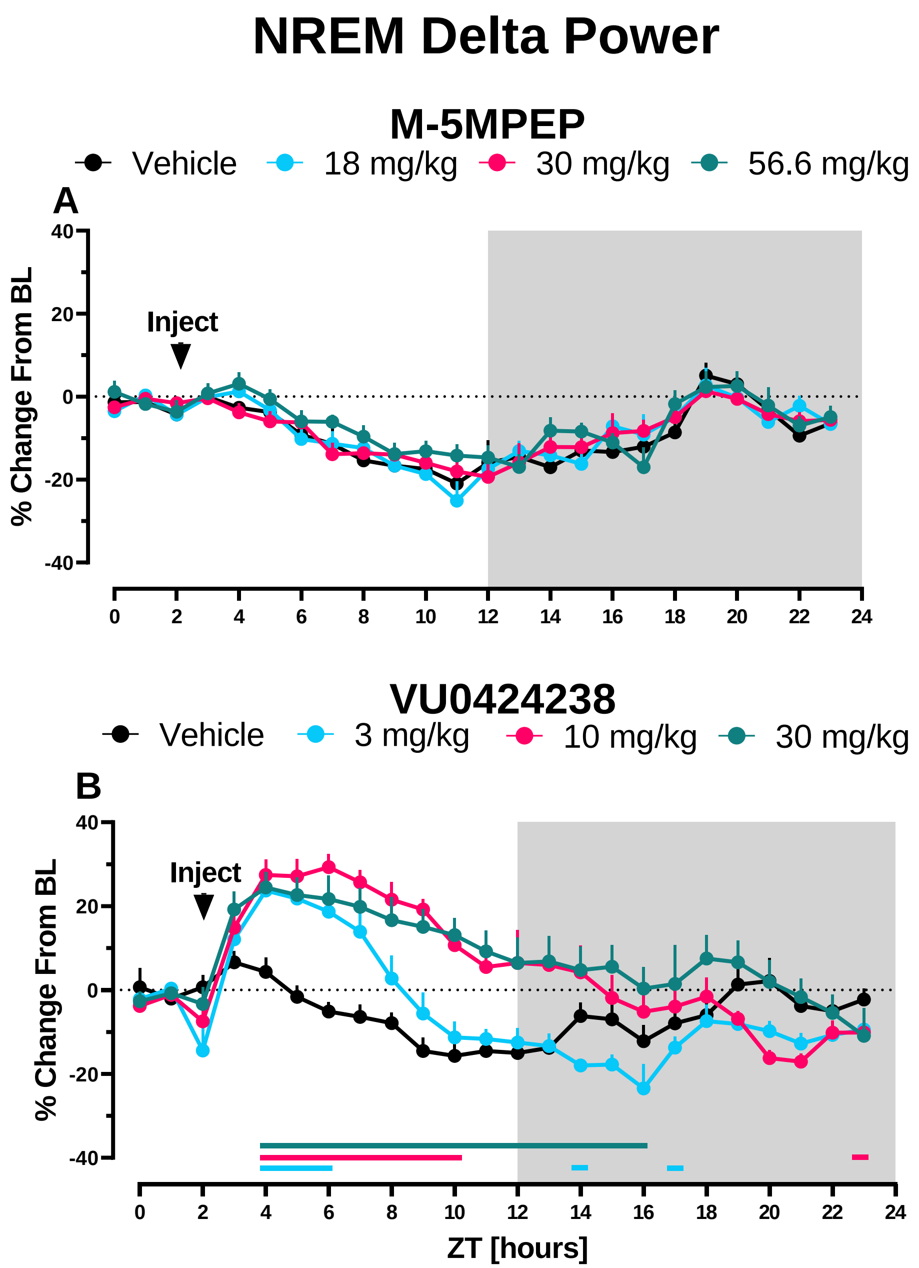

Figure 5. VU0424238 and M-5MPEP had differential effects on delta power during NREM sleep. Data shown are group means ±SEM (n=8) presented in 1 hour bins for effects of M-5MPEP **(A)** and VU0424238 **(B)** on delta power (0.5-4Hz). qEEG spectral power is expressed as the group mean percent change from each individuals averaged 90-minute baseline prior to compound administration. VU0424238 and M-5MPEP were administered at time point 0. Corresponding horizontal colored lines represent time points at which treatment groups were statistically different from respective time points of vehicle-treated group, $p<0.05$.

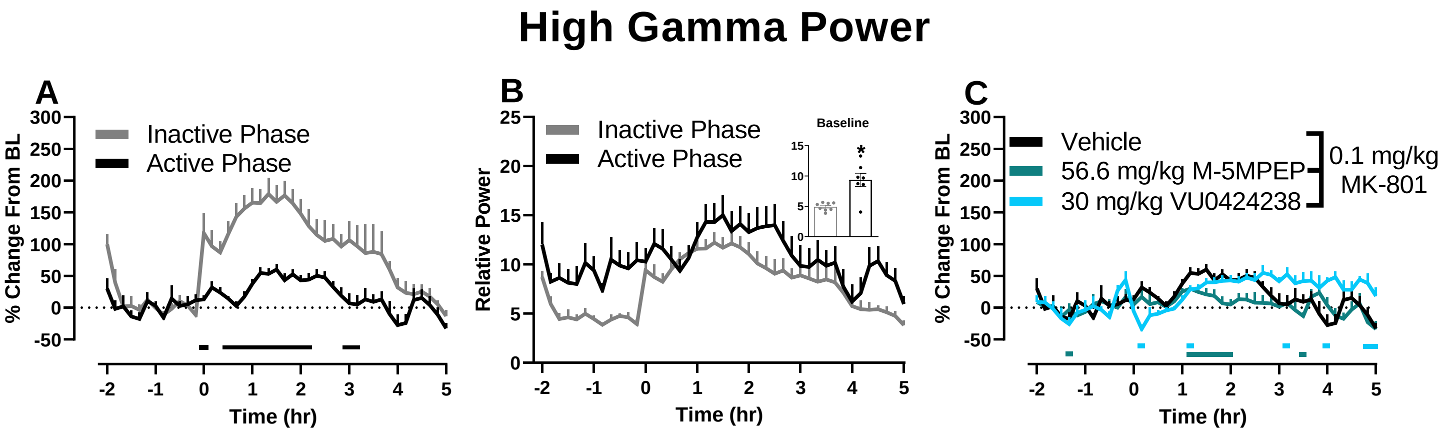

Figure 6. mGlu$_5$ NAM + 0.1 mg/kg MK-801-induced elevations in high gamma power are impacted by light cycle phase. Data shown are group means ±SEM (inactive n=8; active n=7) presented in 10 minutes bins for effects of vehicle combined with 0.1 mg/kg MK801 either during the inactive phase or the active phase. Vehicle was administered at time point 0; MK-801 was administered 30 min after administration of mGlu$_5$ NAMs **(C)** qEEG gamma power is expressed as a percent change from the average of each individual's 90-min baseline just prior to compound administration **(A)** or average relative power across 10-min bins **(B)**. Inset represents the average relative power within the 2 h baseline prior to compound administration **(B)**. qEEG spectral power following administration of mGlu$_5$ NAMs 2 h into the active phase is expressed as a percent change from the average of a 90 minute baseline prior to compound administration **(C)**. Corresponding horizontal lines represent time points at which respective treatment conditions were statistically different from one another **(A,B)** or from vehicle **(C)** $p<0.05$.

Table 1. Statistics for Supplemental Figures 2-6

Frequency/Treatment	Comparison	DF	F or T	P	*	Fig	*Post hoc* results	Significant Time Points (min) or Frequency (Hz)
**M-5MPEP**								
**Delta**	Dose	2.35, 16.48	1.41	0.2735	ns			
**(Wake)**	Time	4.92, 34.42	2.29	0.0681	ns	S2A		
	Dose X Time	6.09, 41.83	0.941	0.4771	ns			
**Theta**	Dose	1.89, 13.23	0.61	0.5493	ns			
**(Wake)**	Time	5.26, 36.80	5.442	0.0006	***	S2B		
	Dose X Time	5.45, 37.43	0.876	0.5136	ns			
**Alpha**	Dose	1.94, 13.58	0.935	0.4136	ns			
**(Wake)**	Time	5.55, 38.86	7.551	<0.0001	****	S2C		
	Dose X Time	6.32, 43.37	1.046	0.4107	ns			
**Sigma**	Dose	1.46, 10.22	2.078	0.179	ns			
**(Wake)**	Time	4.99, 34.91	14.63	<0.0001	****	S2D		
	Dose X Time	6.05, 41.67	1.369	0.2495	ns			
**Beta**	Dose	1.65, 11.55	1.634	0.2359	ns			
**(Wake)**	Time	5.41, 37.85	4.639	0.0017	**	S2E		
	Dose X Time	5.37, 36.89	0.94	0.4717	ns			
**Delta**	Dose	1.78, 12.48	0.668	0.5134	ns			
**(NREM)**	Time	2.70, 18.93	20.84	<0.0001	****	S5A		
	Dose X Time	4.28, 27.11	1.087	0.3851	ns			
**VU0424238**								
**Delta**	Dose	2.046, 14.32	2.332	0.132	ns		Veh v 3.0 mg/kg	
**(Wake)**	Time	4.624, 32.37	4.957	0.0022	**	S3A	Veh v 10 mg/kg	
	Dose X Time	6.138, 41.97	0.0435	0.1228	ns		Veh v 30 mg/kg	
**Theta**	Dose	2.008, 14.06	4.11	0.0392	*		Veh v 3.0 mg/kg	- 30, 160, 210, 240
**(Wake)**	Time	3.787, 26.51	1.982	0.1292	ns	S3B	Veh v 10 mg/kg	220, 270
	Dose X Time	4.885, 31.77	1.459	0.2315	ns		Veh v 30 mg/kg	240, 250, 270
**Alpha**	Dose	2.350, 16.45	31.73	<0.0001	****		Veh v 3.0 mg/kg	10, 30-60, 120-160, 210, 220, 240, 250, 280
**(Wake)**	Time	5.221, 36.55	9.482	<0.0001	ns	S3C	Veh v 10 mg/kg	30-60, 80, 110-140, 160-220, 270, 280, 300
	Dose X Time	6.318, 43.20	2.333	0.0461	*		Veh v 30 mg/kg	30-60, 90, 100, 120-180, 200-220, 240-300
**Sigma**	Dose	2.480, 17.36	25.91	<0.0001	****		Veh v 3.0 mg/kg	20-60, 80-110, 130, 150-190, 210, 220, 250, 260, 280
**(Wake)**	Time	5.123, 35.86	11.6	<0.0001	****	S3D	Veh v 10 mg/kg	30, 40, 60, 80-130, 140-190, 210, 220, 240, 270, 280, 300
	Dose X Time	6.128, 41.90	2.702	0.0254	*		Veh v 30 mg/kg	40-60, 90-130, 160-190, 210, 220, 240-290
**Beta**	Dose	1.926, 13.48	4.229	0.0387	*		Veh v 3.0 mg/kg	110, 220, 250
**(Wake)**	Time	5.095, 35.67	2.092	0.0881	ns	S3E	Veh v 10 mg/kg	0, 170
	Dose X Time	6.089, 41.63	1.64	0.1595	ns		Veh v 30 mg/kg	0, 150, 170, 260-280
**Delta**	Dose	2.07, 14.51	8.737	0.003	**		Veh v 3.0 mg/kg	
**(NREM)**	Time	3.29, 23.04	27.49	<0.0001	****	S5B	Veh v 10 mg/kg	
	Dose X Time	3.95, 26.21	5.317	0.0029	**		Veh v 30 mg/kg	
**mGlu$_5$ NAMs + 0.1 mg/kg MK-801**								
**Delta**	Dose	1.90, 13.33	1.095	0.3597	ns		Veh v 30 mg/kg VU238	- 110, 40, 50, 130-150, 170, 190, 240
**(Wake)**	Time	4.98, 34.85	9.71	<0.0001	****	S4A		
	Dose X Time	5.72, 39.49	2.675	0.0302	*			
**Theta**	Dose	1.91, 13.35	2.965	0.0875	ns			
**(Wake)**	Time	4.32, 30.21	16.81	<0.0001	****	S4B		
	Dose X Time	5.42, 37.33	1.241	0.294	ns			
**Alpha**	Dose	1.61, 11.24	3.657	0.0671	ns		Veh v 30 mg/kg M-5MPEP	20-50
**(Wake)**	Time	2.86, 20.00	6.725	0.0028	**	S4C	Veh v 56.6 mg/kg M-5MPEP	30-100
	Dose X Time	4.90, 33.83	4.698	0.0024	**		Veh v 30 mg/kg VU238	20-60, 80, 130-170, 190-210, 230, 240
**Sigma**	Dose	2.12, 14.83	6.596	0.0082	**		Veh v 30 mg/kg M-5MPEP	90
**(Wake)**	Time	4.33, 30.31	36.28	<0.0001	****	S4D	Veh v 30 mg/kg VU238	190, 230-300
	Dose X Time	5.92, 40.88	2.59	0.0326	*			
**Beta**	Dose	1.61, 11.26	14.49	0.0012	***		Veh v 30 mg/kg M-5MPEP	40-70, 90, 110, 120, 180
**(Wake)**	Time	3.80, 26.59	16.58	<0.0001	****	S4E	Veh v 56.6 mg/kg M-5MPEP	30-50, 70, 90, 200
	Dose X Time	5.31, 36.63	3.961	0.005	**		Veh v 30 mg/kg VU238	160, 170, 190-260, 280-300
**1 Hz bins**	Dose	1.12, 7.84	0.7737	0.4201	ns		Veh v 56.6 mg/kg M-5MPEP	0.5, 7-9, 22-27, 29, 32-36, 45, 47 (Hz)
**(Wake)**	Frequency	1.81, 12.69	32.73	<0.0001	****	S4F	Veh v 30 mg/kg VU238	6, 14-20, 22-26, 48-50, 52-55 (Hz)
	Dose X Frequency	2.21, 15.46	6.149	0.0094	**			
**Inactive v Active High Gamma**	Phase	1, 13	14.86	0.002	**		Active v Inactive	0, 30-130, 180, 190
**Vehicle +0.1 mg/kg MK-801**	Time	42, 539	15.56	<0.0001	****	S6A		
**(Wake, % change)**	Phase X Time	42, 539	5.061	<0.0001	****			
**Inactive v Active High Gamma**	Phase	1, 13	4.417	0.0555	ns			
**Vehicle +0.1 mg/kg MK-801**	Time	4.79, 61.49	11.82	<0.0001	****	S6B		
**(Wake, relative power)**	Phase X Time	42, 539	1.578	0.0135	*			
	Baseline (t-test)	13	4.286	0.0009	***			
**Active Phase High Gamma**	Dose	1.69, 10.12	19.09	0.0005	***		Veh v 56.6 mg/kg M-5MPEP	-80, 70-120, 210
**(Wake)**	Time	4.12, 24.72	4.438	0.0073	**	S6C	Veh v 30 mg/kg VU238	10, 70, 190, 240, 290, 300
	Phase X Time	4.60, 27.20	3.046	0.0289	*			
